# Supplementary material for: Comparison of the human microbiome in adults and children with chronic rhinosinusitis
Source: PLoS One. 2020 Dec 2;15(12):e0242770. doi: 10.1371/journal.pone.0242770 (PMC7710060; doi:10.1371/journal.pone.0242770)
Supplement: S1 Appendix. This appendix contains Fig A–C — (PDF) [file pone.0242770.s001.pdf]

PlosOne Supporting Information S1 Appendix

Article title: Comparison of the Human Microbiome in Adults and Children with Chronic Rhinosinusitis.

Authors: Il-Ho Park, Joong Seob Lee, Joo-Hoo Park, Sung Hun Kang, Seok Min Hong, Il Seok Park, Joo Heung Yoon and Seok Jin Hong

The following supporting information is available for this article:

S1 Table A. Averaged taxonomic compositions in Genus level of Adult group.

S1 Figure A. Species richness indexes.

S1 Figure B. Alpha-diversity indexes.

S1 Figure C. Averaged taxonomic compositions in Species level of adult group and pediatric group.

Table A.

Genus	Proportion
Corynebacterium	0.2507981
Staphylococcus	0.1311886
Haemophilus	0.0604578
Fusobacterium	0.0500684
Mycoplasma_g13	0.0416475
Escherichia	0.0361305
Propionibacterium	0.029079
Anaerococcus	0.0265404
Peptoniphilus	0.0262038
Bacteroides	0.0234132
Moraxella	0.0178917
Streptococcus	0.0141024
Citrobacter	0.0129738
Prevotella	0.0106526
Unclassified in higher taxonomic	0.0006087
ETC(under 1% in average)	0.1781553

Figure A.

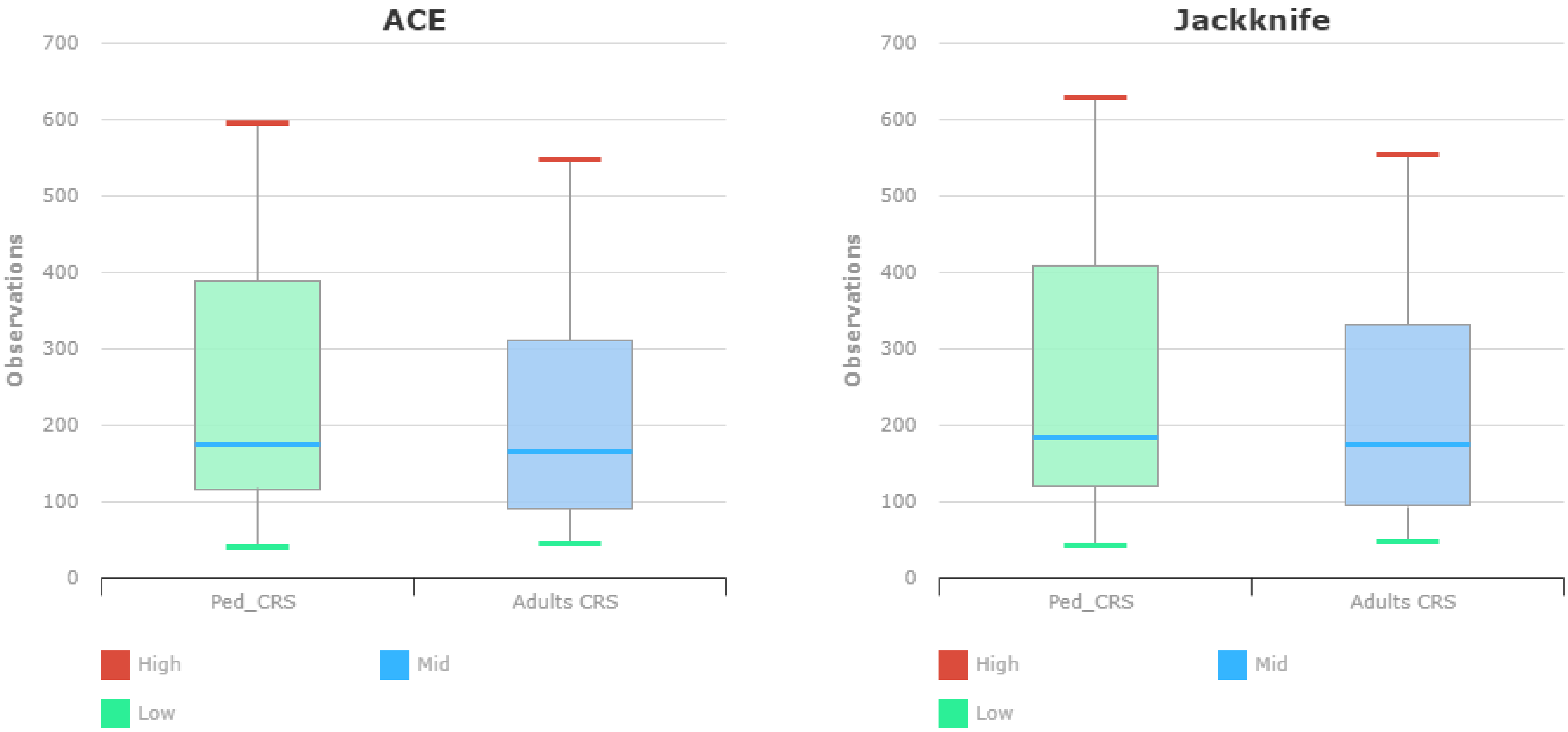
ACE
700
600
500
400
300
200
100
0
Observations
Ped_CRS
Adults CRS
High
Mid
Low
Jackknife
700
600
500
400
300
200
100
0
Observations
Ped_CRS
Adults CRS
High
Mid
Low

Figure B.

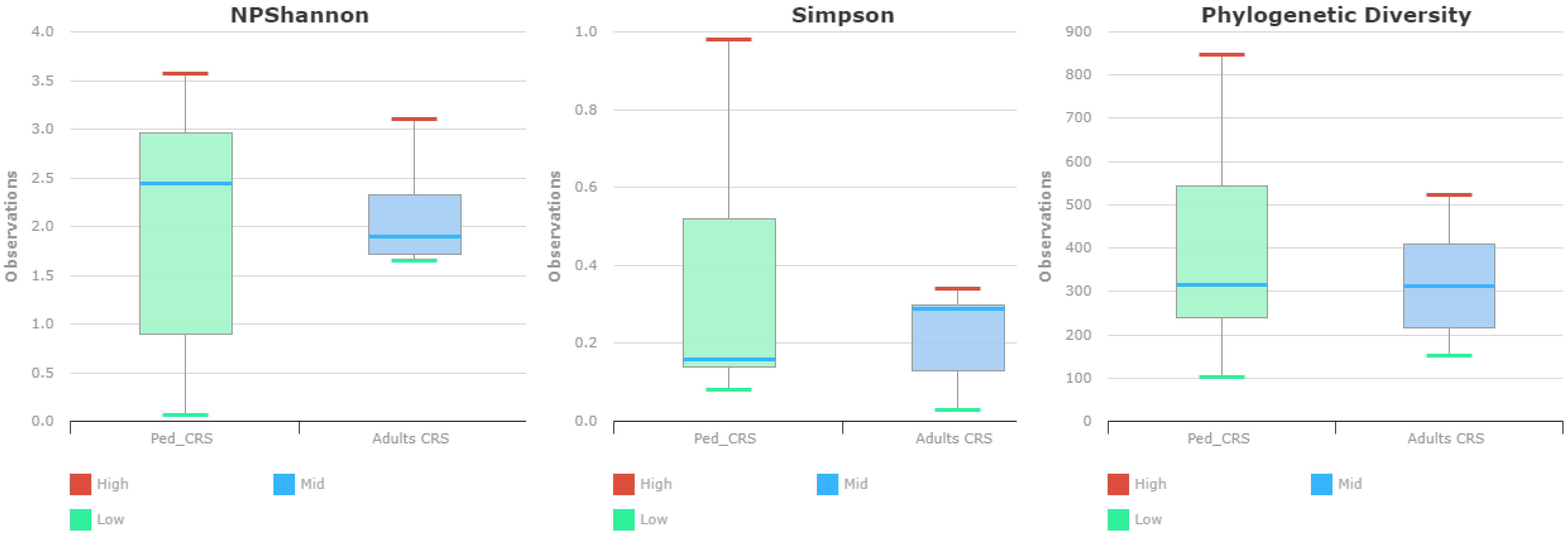
NPShannon
Simpson
Phylogenetic Diversity
Observations
4.0
3.5
3.0
2.5
2.0
1.5
1.0
0.5
0.0
0.8
0.6
0.4
0.2
900
800
700
600
500
400
300
200
100
0
Ped_CRS
Adults CRS
High
Mid
Low

Figure C

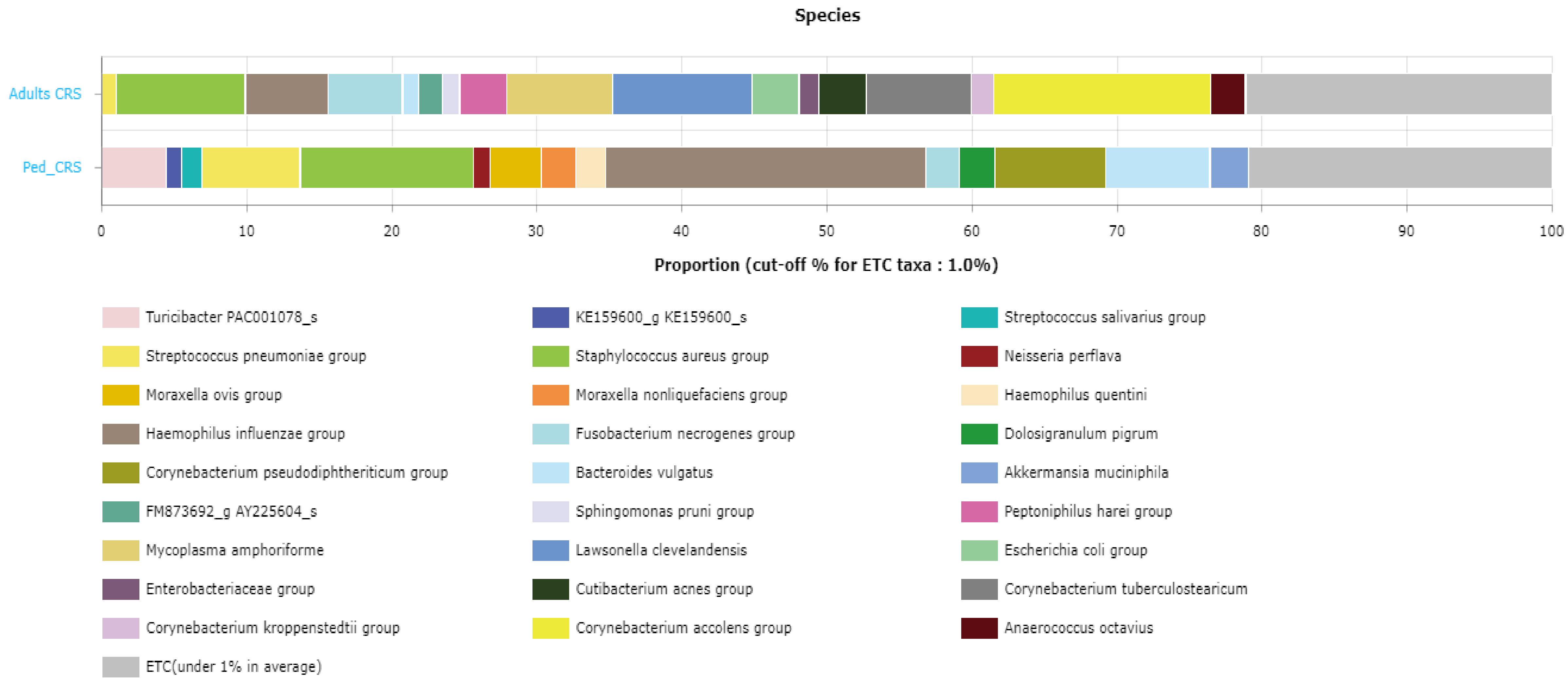

Species
Adults CRS
Ped_CRS
0
10
20
30
40
50
60
70
80
90
100
Proportion (cut-off % for ETC taxa : 1.0%)
Turicibacter PAC001078_s
KE159600_g KE159600_s
Streptococcus salivarius group
Streptococcus pneumoniae group
Staphylococcus aureus group
Neisseria perflava
Moraxella ovis group
Moraxella nonliquefaciens group
Haemophilus quentini
Haemophilus influenzae group
Fusobacterium necrogenes group
Dolosigranulum pigrum
Corynebacterium pseudodiphtheriticum group
Bacteroides vulgatus
Akkermansia muciniphila
FM873692_g AY225604_s
Sphingomonas pruni group
Peptoniphilus harei group
Mycoplasma amphoriforme
Lawsonella clevelandensis
Escherichia coli group
Enterobacteriaceae group
Cutibacterium acnes group
Corynebacterium tuberculostearicum
Corynebacterium kroppenstedtii group
Corynebacterium accolens group
Anaerococcus octavius
ETC(under 1% in average)
